# A Roadmap to the Human Virome

**DOI:** 10.1371/journal.ppat.1003146

**Published:** 2013-02-14

**Authors:** Eric Delwart

**Affiliations:** 1 Blood Systems Research Institute, San Francisco, California, United States of America; 2 Department of Laboratory Medicine, University of California at San Francisco, San Francisco, California, United States of America; Columbia University, United States of America

Low-cost DNA sequencing has greatly democratized genomics, especially for the typically very small genomes of viruses [1]–[3]. The recent acceleration in human virus discovery by metagenomics indicates that many viruses escaped prior detection due to limitations of preexisting technologies. It is now conceivable that all viral species commonly infecting human (i.e., the human virome) will soon be determined. As the value of the human genome and microbiome has become widely recognized, providing crucial reference genomes and opening unanticipated avenues of research, the genetic characterization of the human virome also holds great promises [4].

The human microbiome project, focusing largely on single bacterial cells and metagenomic sequencing of total DNA from feces and other human sites, is unlikely to detect RNA viruses. The generally minuscule size of viral genomes relative to those of their bacterial or eukaryotic hosts also weighs against their easy detection in metagenomic approaches. Viral discovery can be greatly facilitated by simple filtration to enrich the smaller viral particles and by removal of contaminating bacterial and human nucleic acids using nuclease digestion that leave viral nucleic acids protected within their virion shells (Figure 1) [5]. Density gradient ultra-centrifugation has also been used to enrich viral particles.

**Figure 1:**
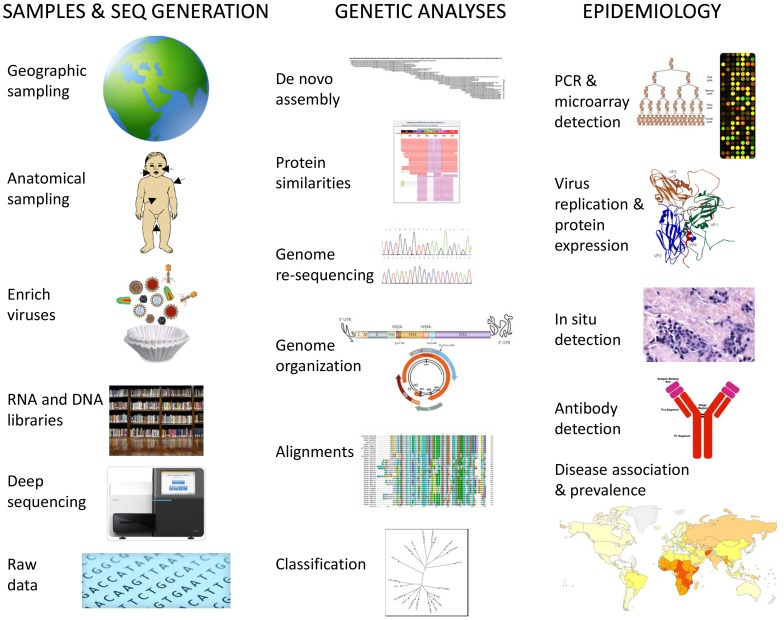
Schematic steps for determination of human virome and its impact on health, including biological samples and data acquisition, genetic analysis, and epidemiology of viral infections.

Knowledge of the human virome will allow the design of sensitive and specific tests to all human viruses using “virochip” microarrays [6] or multiplexed PCR assays (Figure 1). Provided samples from large enough human cohorts are analyzed, it will be possible to determine viral prevalence, likely transmission routes, and most crucially disease association. The development of vaccines and antivirals can then be targeted to the viruses with the largest public health impacts in different geographic regions. Deciphering the complete human virome will therefore improve our understanding, prevention, and treatment of currently unexplained diseases involving viral pathogens, as well as provide insights into the evolution of viral families and cross-species viral transmissions.

Despite the rapid progress being made toward deciphering the human virome, several roadblocks remain to its full characterization and utilization. Below is an abbreviated list of these problems and possible solutions.

## How Much Sampling of the Human Population Is Enough?

An individual's exposure to viruses is influenced by their geographic location, age, lifestyle, and even the season of the year, while their susceptibility to disease is affected by preexisting immunity and both viral and human genetics. Characterizing all human viruses will require casting a very wide nest and analyzing samples collected around the globe from a diverse collection of patients exhibiting a wide range of unexplained symptoms as well as carefully epidemiologically matched healthy subjects. Blood, respiratory secretions, feces, urine, skin swabs, and tissues may all be used for viral metagenomics (Figure 1). Samples likely to yield the most “new” human viruses will likely be from children and immunocompromised patients whose viral loads are expected to be higher and last longer. Subjects living in crowded locations with poor sanitation, nutrition, and healthcare standards are also expected to generally carry a higher viral burden. Sick travelers, exposed to viruses to which they have no preexisting immunity, may also be rich sources of “new” viruses.

The constituent species of the human virome will also vary over time due to ongoing zoonotic transmissions from animals whose own viromes will also be changing [7]. Analyses of humans with extensive contact with wild or domesticated animals such as bush-hunters, abattoir workers, or those heavily exposed to insect bites in regions of high biodiversity will increase the odds of detecting new human viruses [8].

When will the human virome be completed? When, despite geographically diverse and intense sampling of heavily exposed and susceptible populations, the rate of “new” human virus discovery falls to near zero, the human virome (at least as it exists at that time) may be considered near completion. The ongoing exchange of viruses between host species will require ongoing surveillance for zoonotic outbreaks of emerging viral pathogens.

## Which “New” Viruses Are Human Pathogens?

In order to acquire maximum value from the human virome, the role of newly characterized “orphan” viruses in causing unexplained human diseases will need to be determined [9]. Comparing viral incidence (nucleic acids or IgM) or prevalence (IgG) in disease versus matched healthy controls is a powerful approach. The collection of samples from unexplained symptomatic cases and healthy controls (epidemiologically matched for age, sex, location, and sociodemographics/lifestyles) will be useful for both disease association studies and as input material for further viral discoveries. Provided longitudinally collected plasma/serum or biopsies are available, sero-conversion or the detection of antigens/nucleic acids in affected tissues will help ascertain the pathogenicity of new viruses (Figure 1). For many viruses, rigid compliance to Koch's postulates to ascertain pathogenicity remains unlikely and relaxed criterion might be acceptable [10]. Large-scale antibody sero-prevalence studies with ELISA or antigens micro-arrays [11] using voluntary donations to blood banks worldwide may be used to efficiently determine exposure in different geographic regions. Serum from different age cohorts will help determine age of initial exposure.

The high prevalence in human plasma of chronic viral infections for whom there are currently no disease association such as anelloviruses (e.g., TTV) and the flavivirus GBV-C, both among the earliest viruses identified by strictly molecular methods, indicates that the continued identification of human viruses causing low, rare, or no pathogenicity can be expected [12]. Generally commensal and highly prevalent infections may become pathogenic in the context of immunodeficiency, co-infections, or in human hosts with rare genetic susceptibility [13]. Generally harmless infections inducing severe clinical symptoms in rare patients may reveal low-frequency human susceptibility alleles of utility for targeted vaccination or surveillance [12]. The very high genetic diversity of some viral families such as the *Anelloviridae* or *Picornaviridae* certainly complicate studies of their pathogenicity as this may vary widely between close variants of the same species, as also seen for example between the numerous serotypes of enteroviruses or types of papillomaviruses [14].

## Can Highly Divergent Viral Families Be Identified?

Identifying “new” viruses through protein sequence similarities to those of already known viruses has proven its utility [15], [16]. Unfortunately, in silico protein sequence similarity search methods such as BLASTx will not recognize viral families that do not already have a related genome in public databases or do not encode widely used viral hallmark protein such as the RNA-dependent RNA polymerase (RdRP) [17], [18]. Sequences from genetically uncharacterized and highly divergent viral families will therefore be classified as being of unknown taxonomic origin. Until prototype genomes of these viral families (from any biological sources including animals and environmental sources) are annotated as viral and submitted to public databases these “BLAST resistant” viruses will remain difficult to identify by sequence similarity-based approaches.

Several approaches may help identify such viral genomes. The repeated detection of the same unclassifiable sequence in multiple subjects with similar symptoms may indicate the presence of a novel viral family and provide genetic foothold sequences that can be extended in silico as the nucleus of larger contigs (overlapping contiguous sequences) or by laboratory means. Furthermore if viral genomes are enriched relative to contaminating host cell or bacterial DNA, randomly generated sequence reads are expected to more easily assemble into overlapping sequences and form contigs than reads from the much larger cellular genomes. Long contigs of unknown taxonomic origins derived from viral-genome-enriched nucleic acids mixture may therefore include viral segments from still uncharacterized viral families. Further improvement in the sensitivity and speed of bioinformatic methods to detect very weak viral protein motifs will also facilitate detection of highly divergent viviral sequences. Candidate viral ORFs may also be identified through in silico–predicted protein folding to reveal similarities to common viral structures such as the capsid jelly-roll.

Traditional approaches of virus detection such as the induction of cytopathic effects following inoculations of cell lines may also provide starting material for sequencing of viral genomes with little or no sequence similarities to the currently characterized animal virome. Expanding genome characterization of highly divergent viruses infecting diverse cellular hosts or found in environmental samples enriched for viruses such as sewage should facilitate the recognition of currently BLAST-resistant human viruses.

Confirming the nature of highly divergent tentative novel viral families identified strictly by metagenomic means may involve genome copy number amplification or antigen expression following cell or animal inoculations, detection of host antibodies to putative viral antigens, and/or generation of viral-like particles by overexpression of their hypothetical capsid proteins (Figure 1).

## Can the Viral Host Be Identified from Metagenomic Data?

Finding a virus in feces or respiratory fluids does not guarantee that a virus replicates in this host's cells as it may be ingested from a dietary source or inhaled [19]. Viruses may also be replicating in bacteria or parasites such as protozoan and nematodes [20]. Testing the human tropism of “new” viruses may be done by measuring antibody response or amplifying the virus in vitro using human cells. The detection of viral antigens or nucleic acids in internal sites such as blood, CSF, or tissues may also be interpreted as supporting evidence for replication in this host's cells.

In silico approach to infer the phylum of the likely host of a viral genome involves the analysis of their di- and tri-nucleotides composition. After machine training with a set of distantly related viral genomes in the *Picornavirales* order (positive single-stranded picorna-like viruses), discriminant analysis was able to differentiate between viruses infecting plants, insects/nematodes, or vertebrates [21]. Such DNA signatures may be used to narrow the range of possible hosts for viruses of uncertain origins.

Reagents used for making libraries for deep sequencing may also be contaminated with reverse transcriptase sequences, and densovirus and circovirus-like sequences have been reported in nucleic acid purification columns [22]. Contamination with DNA from prior libraries may also be a significant problem in high-throughput sequencing platforms capable of generating billions of sequence reads. Carefully ruling out contamination by re-extraction of the original biological material or of freshly collected samples using an alternative method and confirmatory PCR under contamination-free conditions is highly desirable [23].

## The Human Virome in the Future?

The number of ICTV-approved viral families infecting eukaryotes is significantly greater than those infecting the more ancient and diverse prokaryotes. Approximately a third of eukaryotic viral families include species known to infect humans, also likely reflecting a sampling bias in favor of eukaryotic hosts, particularly humans [14]. Numerous recent metagenomics and consensus PCR studies have expanded the number of genera and species infecting human particularly, but by no means exclusively, in the *Astroviridae*, *Parvoviridae*, *Picornaviridae*, and *Polyomaviridae* families. As more people, from more wide spread geographic areas and exhibiting different symptoms, are sampled, the number of viral species, genera, and possibly even families shown to infect humans will continue to increase.

A significant fraction of mild to extremely severe symptoms of likely infectious origin remains unexplained in both developed and developing countries including respiratory problems, diarrhea, and encephalitis [24], [25]. Auto-immune diseases such as diabetes may also be triggered by unknown viral infection, and carcinogenic human viruses may still remain uncharacterized [26]. Judiciously collected samples from patients with these varied conditions are likely to be fertile ground for further viral pathogen discoveries and/or for assigning new pathogenic potential to already known viruses. Outbreaks of unusual symptoms in humans will also continue to yield numerous “new” emerging human viruses mostly originating from animal sources.

As a result of more sensitive molecular methods, an increasing number of asymptomatic infections are also being recognized [27]. Although a majority of the still uncharacterized human virome is likely to be pathogenic in only a small fraction of its human hosts, their very high prevalence may still translate into a significant health burden.

The availability of simple, open source software for effective de novo assembly and similarity searches optimized for viral discovery capable of handling an increasing data flow will greatly facilitate the entry of new groups into the field. Frequently updated and curated databases of complete and partial viral genomes that cover the full extent of known viral diversity, while minimizing redundancy of closely related variants and mislabeled sequences, will also accelerate new virus identification.

Animal virus discovery is also poised for rapid advances and because disease causation can be more easily determined may rapidly lead to improved diagnostics, effective transmission control methods, and vaccinations. Characterizing more of the animal virome, whose diversity will dwarf that of human, will help identify the origins of many currently human viruses and the potential sources of future zoonoses. Given the recent expansion of arthropod-borne viruses infecting human and animals, the inoculation of mammalian cells with insect pools is likely to reveal numerous arboviruses with potential human tropism.

Mining of eukaryotic genomes for viral sequences has revealed numerous viruses integrated into host chromosome germlines and the minimum age of many viral families [28]. Such endogenized genomes can also provide sequences to search for related exogenous viruses. The mining of cancer transcriptomes for viral sequences may also reveal novel cancer-associated viruses [29].

While the human virome is a moving target due to zoonoses, the rate at which such animal viruses adapt to human–human transmission is unclear [7], [8]. Besides such infections, there remains already circulating and highly prevalent human viruses still to be characterized [30]. Characterizing the human virome in combination with studies of their prevalence and disease association will provide a better understanding of which viruses account for the large diagnostic gap seen for many diseases of possible infectious origins. Measuring the impact of these infections on public health will allow more informed decision about which viruses to target for behavioral, environmental, vaccine, or pharmaceutical interventions. The highest hurdle to characterizing the human virome and deciphering its biology will likely be the collection of sufficient numbers of biological samples and their epidemiological metadata from sick and healthy individuals world-wide for both metagenomics-based discovery and sufficiently powered disease association studies.
